# Deadly, Dangerous, and Decorative Creatures

**DOI:** 10.3201/eid2802.AC2802

**Published:** 2022-02

**Authors:** Byron Breedlove

**Affiliations:** Centers for Disease Control and Prevention, Atlanta, Georgia, USA

**Keywords:** art science connection, emerging infectious diseases, art and medicine, about the cover, Deadly, Dangerous, and Decorative Creatures, Gilded Vectors of Disease, P.R. Morley Horder, public health, vector-borne infections, transmission, mosquitoes, Aedes spp., Anopheles spp., ticks, fleas, bacteria, viruses, Plasmodium spp., parasites, protozoa, malaria, chikungunya, West Nile fever, yellow fever, Zika virus, dengue virus, ticks, Lyme disease, ehrlichiosis, babesiosis, anaplasmosis, Rocky Mountain spotted fever, Crimean-Congo hemorrhagic fever, Heartland virus disease, plague, murine typhus, body louse, louse-borne relapsing fever, Tsetse fly, African trypanosomiasis, sleeping sickness, sandflies, leishmaniasis, small mammals, rodents, hantavirus infection, rat-bite fever, Lassa fever, salmonellosis, bedbug, zoonoses, London School of Hygiene & Tropical Medicine

**Figure:**
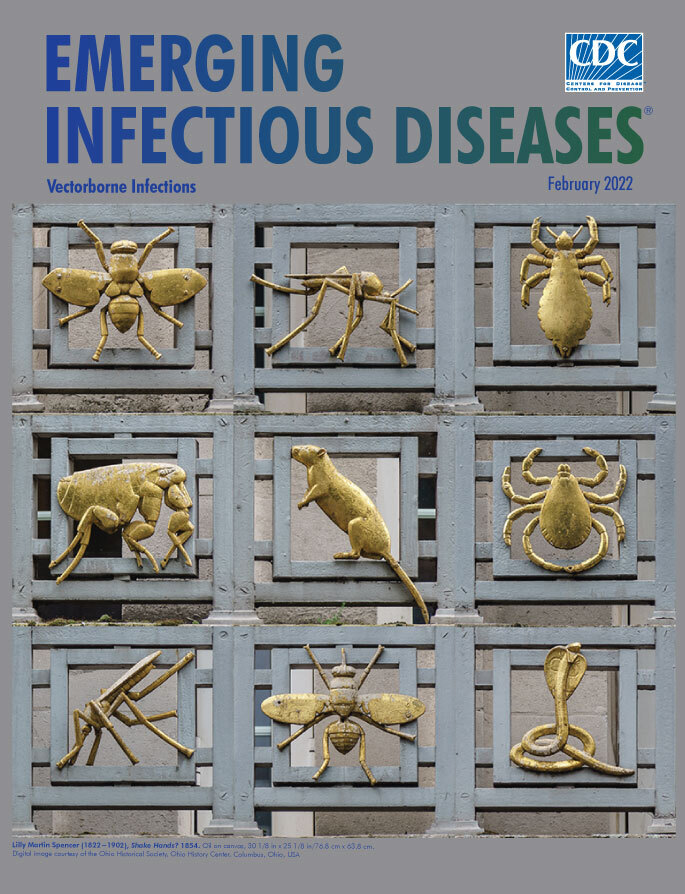
**P. R. Morley Horder, designer (1870−1944); J. Starkie Gardner Ltd, fabricator (years active 1884−1976). Gilded Vectors of Disease, 1912**. Gilded bronze and iron railings. 10.2 in x 10.6 in/26 cm x 27 cm. Images courtesy of Library & Archives Service, London School of Hygiene & Tropical Medicine, London, United Kingdom.

According to the World Health Organization, vectorborne diseases account for more than 17% of all infectious diseases, lead to more than 700,000 deaths annually, and contribute to a large overall global burden of debilitating disease. Mosquitoes, ticks, and fleas are among the arthropods responsible for transmitting many of the myriad bacteria, viruses, and parasites that cause vectorborne diseases―as are some mammals. 

In terms of infectious disease transmission, mosquitoes are considered the most dangerous animals on earth. They are vectors that can spread the *Plasmodium* parasites that cause malaria, as well as chikungunya, West Nile, yellow fever, and Zika viruses, plus 4 types of dengue virus. Ticks, responsible for causing most vectorborne diseases in the United States and Europe, are vectors for agents causing Lyme disease, ehrlichiosis, babesiosis, anaplasmosis, Rocky Mountain spotted fever, Crimean-Congo hemorrhagic fever, and Heartland virus disease. Fleas carry pathogens that cause diseases such as plague and murine typhus. Other vectors include the body louse, which spreads louse-borne relapsing fever; the tsetse fly, which transmits African trypanosomiasis (sleeping sickness); and sand flies, which transmit the pathogen that causes leishmaniasis. Small mammals, particularly rodents, are vectors for the agents of plague, hantavirus infection, rat-bite fever, Lassa fever, and salmonellosis.

This month’s cover features a collage of images arrayed like mug shots for a number of these creatures responsible for many vectorborne diseases across the globe. To many involved in the disciplines of public health and infectious disease, these images will be familiar as the “Gilded Vectors of Disease,” an ornate, Art-Deco bestiary of sculpted bronze figures bracketing each side of 15 iron balconies located across the front and sides of the London School of Hygiene & Tropical Medicine. Each of these relatively small sculptures, measuring approximately 26 cm × 27 cm, appears three times across the various balconies and is bolted onto an iron square that encompasses much of the vector’s body. Various wings, legs, and tails that extend in all directions help distinguish one vector from the other. 

The bronze figures included in the cover collage are seven arthropods―six insects and one arachnid―one mammal, and one reptile (Figure). The insects are an *Aedes* mosquito, *Anopheles* mosquito (the one with its tail pointing upward), body louse, flea, tsetse fly, and housefly; the arachnid is a tick, and the mammal a rat. The snake, an Indian cobra, was included because it makes the cover image symmetrical, and snakes and other reptiles carry a range of pathogens including bacteria, viruses, parasites, and worms. Although reptiles are not considered disease vectors, their possible role as reservoirs of zoonotic parasites has garnered new attention among some researchers, including Mendoza-Roldan et al. 

**Figure:**
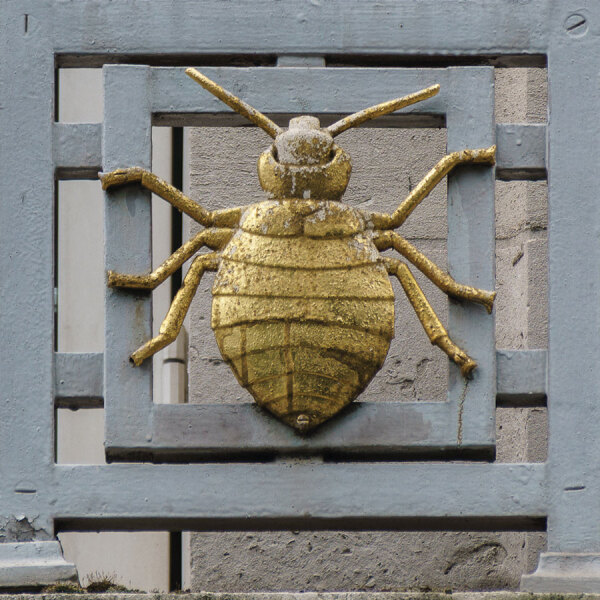
Missing from the cover collage of the bronze figures is this bedbug. Although a worldwide problem, bedbugs are not a disease vector.

Precise details on whether an individual or a committee approved the creation and installation of the 10 animals that festoon the school’s balconies are lost to history. Researcher and writer Ann Datta notes, “The actual selection process that led to the particular choice of animals in the frieze is unknown.” She recounts that during 1926−1928, architects Percy Morley Horder and Verner Rees designed “the steel-framed building with a Portland stone façade” and that Horder was responsible for designing the frieze. The iron balconies, and presumably the bronzed creatures, were the handiwork of J. Starkie Gardner Ltd., Decorative Metal Workers, from the Southfields district of inner London (the firm ceased operations in 1976). 

Datta states that “All the animal figures are somewhat stylized although most retain the essential characters for scientific identification to genus level.” Created nearly a century ago, the Gilded Vectors of Disease are enduring symbols of the importance of national and international public health efforts to detect, understand, prevent, and track vectorborne diseases.
